# Supplementary material for: Seed Transmission of Three Viruses in Two Pear Rootstock Species Pyrus betulifolia and P. calleryana
Source: Viruses. 2022 Mar 14;14(3):599. doi: 10.3390/v14030599 (PMC8949422; doi:10.3390/v14030599)
Supplement: Supplementary file 1 [file viruses-14-00599-s001.zip › viruses-1584100-supplementary/Supplementary files/Supplementary Figure.pdf]

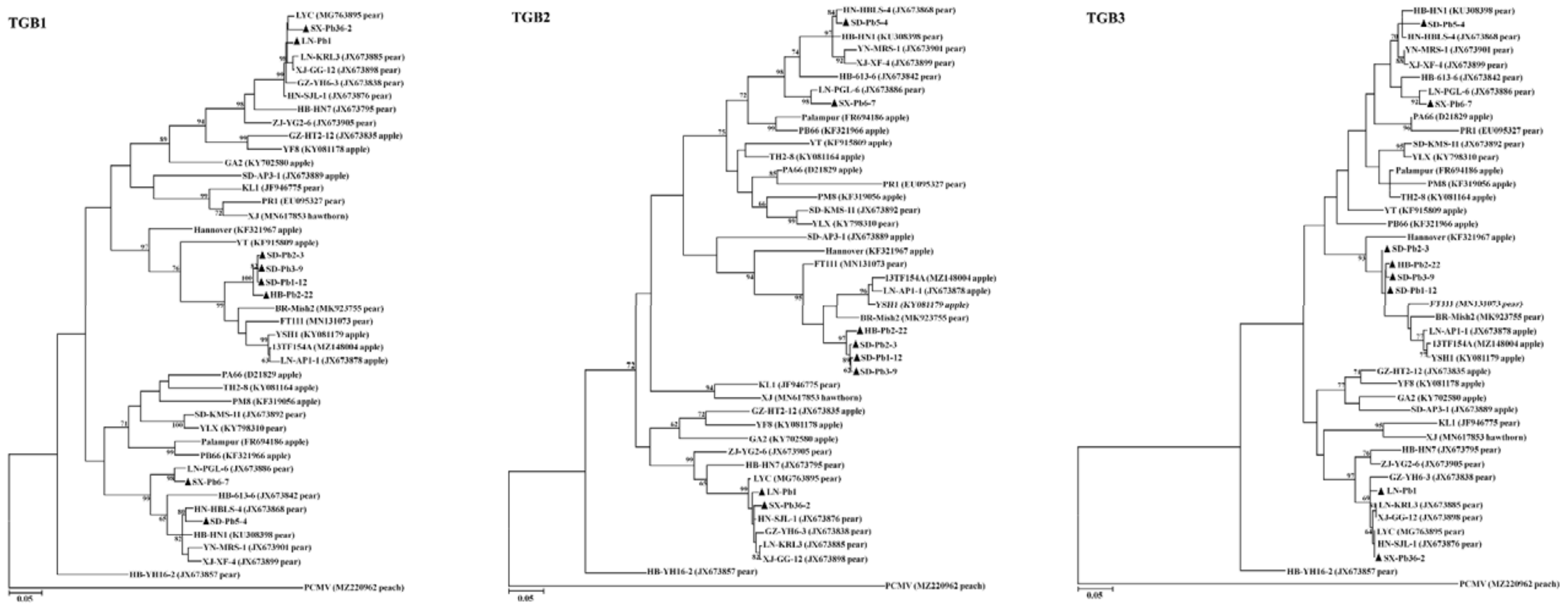

Figure S1. Unrooted maximum-likelihood (ML) phylogenetic trees generated from the nucleotide sequences of TGB1, TGB2 and TGB3 genes of apple stem pitting virus (ASPV). Each reported sequence was marked by its isolate name followed by a GenBank accession number and a host name. The sequences determined in this study were highlighted by black triangles and marked by their geographic origins, hosts and clone IDs, and sequences from seeds were identified with ‘Z’. The corresponding sequence of peach chlorotic mottle virus (PCMV) was used as an outgroup in each phylogenetic tree..
