# Supplementary material for: Seed Transmission of Three Viruses in Two Pear Rootstock Species Pyrus betulifolia and P. calleryana
Source: Viruses. 2022 Mar 14;14(3):599. doi: 10.3390/v14030599 (PMC8949422; doi:10.3390/v14030599)
Supplement: Supplementary file 1 [file viruses-14-00599-s001.zip › viruses-1584100-supplementary/Supplementary files/Supplementary Tables.pdf]

Table S1. Primers designed for amplification of ASGV, ACLSV and ASPV genes

Virus	Primer	Sequence (5′-3′) [a]	Target gene	Position (nt) [b]	Product size (bp)
ASGV	MP-F	GGGTCMGAATGGCWATCGTC	MP	4781-4800	1003
	MP-R	TGCCTTCAAAGCTTTCACCTTC		5762-5783	
	CP-F	GAGTTTGGAAGACGTGCTTCAACAAGCG	CP	5644-5671	713
	CP-R	CCTAACCCTCCAGTTCCAGGTTACTCTCC		6328-6356	
ACLSV	CP-F	ATGGCAGCAGTTCTGAATC	CP	6769-6787	582
	CP-R	CTAGATGCAAAGATCAG		7334-7350	
ASPV	90-110-F	CCTTATTACCACCCATTAGGT	CP	7830-7850	1366
	1500-1520-R	GGGATCAACTTTACTAAAAGCAT		9173-9195	
	TGB- F	GTGTGTAAGCATATTAGG	TGB	6658-6675	1203
	TGB-R	CTACACCCTAACCTAATG		7843-7860	

[a] M:A/C, W:A/T. [b] Target position corresponding to ASGV (KU198289.1), ASPV (JF946775.1) and ACLSV (KY310577.1) genome sequences.

Table S2. Primers designed based on RNA-Seq derived contigs for RT-PCR amplifications of ASPV genome

Segment	Primer	Sequence (5´-3´)	Position (nt)[a]	Product size (bp)
S1	F1	GATACGCAAACAAACTCTGAACAA	1-24	618
	R1	AGAATGTTATTAGAGCCTCCTTAG	618-641	
S2	F2	GAATCATACGACCATCAGTTCCAC	1514-1537	1644
	R2	GAGCAGCTTGTTATCTTCCACCTT	3157-3180	
S3	F3	AGGAAATTAATAATGATTCTTGGG	4160-4183	640
	R3	CAAATGAGCTGCCACAATCTTCTT	4790-4813	
S4	F4	TCACGGAGATTGCTTGAGGGCCCA	6991-7014	760
	R4	GACACAACCTACTATTGAAACTGA	7750-7773	
S5	F5	GTTGAAAGCTCTGCATCACTAGAA	8936-8959	311
	R5	GAAAATCTAGTTAAAACAAAAATA	9246-9269	

[a] Target position corresponding to the genome sequence of ASPV isolate DL detected in this study.

Table S3. Mixed infection of viruses ASGV, ACLSV and ASPV in seeds and field seedling plants of *P. betuleafolia* and *P. calleryana* tested by nmRT-PCR

Sample	No. of tested sample	No. of mixed infection samples			
		ASGV/ACLSV/ASPV	ASGV /ASPV	ASGV/ACLSV	ACLSV/ASPV
Seed	582	1	11	5	4
Seedling	877	0	6	5	1

Table S4. Contigs assembled from RNA-seq derived reads

Contig ID	Size (nt)	Position (nt)	Identity (%)	Matched sequence
c4204	1751	10-1759	97.77	MG763895.1
c9611	392	4073-4464	97.70	KU798310.1
c923	964	1880-2843	97.32	MG763895.1
c14232	1135	3098-4232	97.97	MG763895.1
c702	2695	4482-7176	97.37	MG763895.1
c2698	2003	7200-9204	97.70	MG763895.1
